# Climate Change Impact: The Experience of the Coastal Areas of Bangladesh Affected by Cyclones Sidr and Aila

**DOI:** 10.1155/2016/9654753

**Published:** 2016-10-27

**Authors:** Russell Kabir, Hafiz T. A. Khan, Emma Ball, Kay Caldwell

**Affiliations:** ^1^Department for Allied and Public Health, Faculty of Medical Sciences, Anglia Ruskin University, Bishop Hall Lane, Chelmsford, Essex CM1 1SQ, UK; ^2^School of Health Sciences, Faculty of Health, Education and Life Sciences, Birmingham City University, Birmingham B15 3TN, UK; ^3^The Oxford Institute of Population Ageing, The University of Oxford, 66 Banbury Road, Oxford OX2 6PR, UK; ^4^Department of Product Design and Mathematics, School of Science and Technology, Middlesex University, London NW4 4BT, UK; ^5^Institute of Nursing and Midwifery, School of Health and Education, Middlesex University, London NW4 4BT, UK

## Abstract

Bangladesh is considered one of the countries most at risk to the effects of climate change and its coastal area is most vulnerable. This study tries to explore the experiences of cyclones Sidr and Aila affected people living in the coastal areas of Bangladesh. This study was conducted in the cyclone Sidr affected Amtali Upazila of Barguna District and in the cyclone Aila affected Koyra Upazila of Khulna District. Primary data collection was done using Focus Group Interview and then a thematic analysis approach was used for analysis. Three core themes emerged from the analysis and they are, firstly, impacts of climate change on the socioeconomic condition of the people, secondly, the impact on the health status of the population, and finally the impact on vulnerable people. Findings show that the effects of climate change have serious consequences on the livelihood patterns of the affected population and on their overall health status. As a result, the unfavorable health condition of these affected people makes them more vulnerable to various emerging diseases.

## 1. Introduction

Climate change is perhaps the most widely discussed issue among the recent global environmental changes and research studies are showing that it links to natural disasters that are affecting the social and economic wellbeing of populations [1]. A study by Wei et al. [2] among the Centres for Disease Control & Prevention health professionals in Shanxi Province in China reveals that climate change is happening at both global and local levels and would lead to adverse impacts. They also noted that agricultural production, population health, and natural ecology had already been affected by climate change in their justification with more extreme weather events. This research finding also shows that coastal population financial life has been affected by climate change and due to a lack of job opportunities and to maintain the household expenses, some of the families and in some cases the heads of the households are leaving the village and migrating to different cities. Similar findings are shown in another study by Guha-Sapir et al. [3] where Tsunami affected unemployment in Tamil Nadu. Climate change affected the livelihood pattern and job security of fishermen in Coromandel Coast of New Zealand [4].

The impact of climate change will be felt by different parts of the world and by different people; poor countries like Bangladesh are going to be worst hit. For example, research by Furberg et al. [5] on Sami population shows that rapidly changing unstable weather patterns affect their living patterns. Furthermore, studies by Adebo and Sekumade [6], Adeniyi et al. [7], and Guha-Sapir et al. [3] suggest that women and children tend to be the worst affected. Additional studies by Devkota et al. [8], Kendrovski and Spasenovska [9], Davies et al. [10], and Bhuiyan and Khan [11] also show how these groups are suffering from health problems because of climate change. In disaster times, children and babies lack the capacity to escape from the hazard. In a study of 1991 Bangladesh cyclone, for example, children and older people died more disproportionately than others in the population [12]. About 71% of respondents mentioned that children suffer most due to the adverse effects of climate change and this was closely followed by elderly people and women at 63%, respectively [13]. This shows that women are the most vulnerable group who are the worst victim of the climate change followed by children and the elderly population. Other pieces of research show that the elderly populations suffer the most adverse health conditions due to changes in weather conditions Grundy [14], Hansen et al. [15], Mortreux and Barnett [16], and Filiberto et al. [17].

The gradual rise of average air and oceanic temperatures will change the rainfall and snowfall patterns, cause droughts and heat waves, intensify tropical cyclones and floods, and increase sea levels. Bangladesh is considered to be highly vulnerable in the context of climate change. It is frequently at the mercy of the forces of nature, especially water from the sky, land, and sea [19].

The climatic conditions of Bangladesh are influenced by a number of global and regional scale factors. These factors include geographical location, the effect of North-South continental scale atmospheric pressure gradient (terrestrial to oceanic), the influence of the jet stream stretched from South East Asia to Northern Africa on the monsoon wind system, changes in the solar albedo due to land use, land cover change in the region and its impacts on wind pattern, and fluctuations in the terrestrial and sea surface temperature [20]. Bangladesh is already evidencing the adverse impacts of global warming and climate change. The following impacts have been observed: hotter summers, irregular monsoons, untimely rainfall, heavy rainfall over short periods (causing water logging and landslides), very little rainfall in dry periods, increased river flow and inundation during monsoon, increased frequency, intensity, and recurrence of floods, crop damage due to flash floods and monsoonal rain, crop failure due to drought, prolonged cold spells, salinity intrusion along the coast (leading to scarcity of potable water and redundancy of prevailing crop practices), coastal erosion, river bank erosion, deaths due to extreme heat and cold, increasing mortality and morbidity, and prevalence and outbreak of dengue, malaria, and diarrhoea [21].

In recent years, Bangladesh was hit by two consecutive cyclones* Sidr* in 2007 and* Aila* in 2009. Paul [22] found that cyclone* Sidr* that hit Bangladesh on 15th of November 2007 caused about 3,406 deaths and over 55,000 people sustained physical injuries. Heavy rain accompanying cyclones and tidal waves due to wind effects caused extensive physical destruction, casualties, damage of crops and livestock, and flooding in a total of thirty districts across the South Western coastal district of Bangladesh [23]. Cyclone* Sidr* affected nine districts of Bangladesh. The most devastated districts were Bagerghat, Barguna, Patuakhali, and Pirojpur [24].

After Sidr, the Government of Bangladesh [18] carried out a rapid initial assessment of the damage. Their assessment found a widespread outbreak of waterborne disease, respiratory tract infection (RTI), and other related infections. People in the nine surveyed areas were at risk of communicable diseases: diarrhoea, dysentery, acute respiratory infection, and pneumonia, and children aged five years or younger were vulnerable.

Cyclone Aila hit the southern coastline of Bangladesh hard on 25th of May 2009. It was really a unique event as a storm like this had not hit the Sundarbans within the last three decades [25]. Satkhira and Khulna districts of Bangladesh suffered the heaviest damage along with Bagerhat, Pirojpur, Barisal, Patuakhali, Bhola, Lakshmipur, Noakhali, Feni, Chittagong, and Cox's Bazar [26]. There had been an outbreak of diarrhoeal disease when cyclone* Aila* hit coastal areas of Khulna as an acute scarcity of drinking water and food worsened the sufferings of thousands. Although no official data were available on the diarrhoeal deaths, an approximate figure of 15 deaths was reported by the locals in Koyra, Paikgacha, and Dacope [26].

There is growing scientific evidence from the literature that a changing climate is responsible for influencing all kinds of natural disasters and these natural disasters are affecting human lives. This study tries to explore the experiences of cyclones Sidr and Aila affected people living in the coastal areas of Bangladesh.

## 2. Methodology

The study was conducted in the South West part of Bangladesh. The data collection process started in December 2011 and lasted for about a month. The two most vulnerable coastal districts, namely, Barguna and Khulna, were selected. The administration of Bangladesh is divided into several hierarchal units and these units are division, district, upazila, and union. Barguna was the worst hit district by cyclone Sidr (2007). Most of its flood ridges were washed away and people are faced with the daily difficulty of tidal seawater engulfing their land [27]. Baliatali village and Ghopkhali village of* Amtali Upazila* of Barguna were selected for data collection. Khulna District was worst damaged by cyclone Aila (2009). In Khulna District, Aila has hit 6 upazilas out of 9. Reports stated that 545,954 people were affected in the district, which includes 120,203 families. According to data, Koyra was the most affected upazila by cyclone Aila [26].

**Figure 1:**
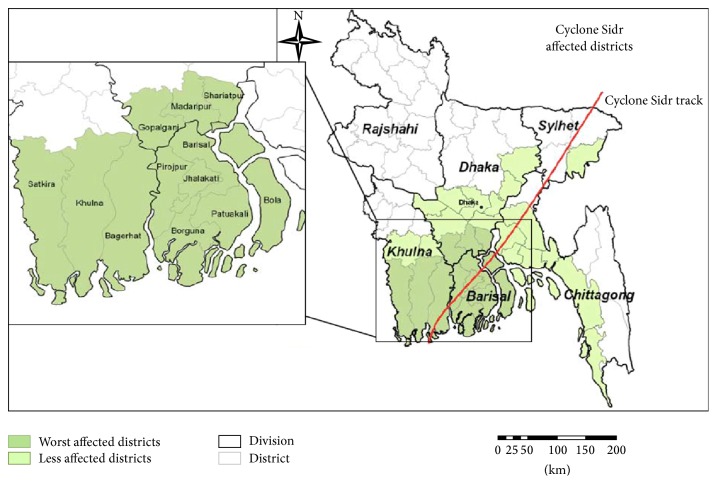
Districts affected by cyclone Sidr. Source ([18]: 4).

Several government and nongovernment organizations were working in the affected area. For each focus group discussion (FGD) with the knowledgeable people, a maximum of 10 persons, one from each organization working in the affected area, were selected.  Figure 1 shows that two FGDs were conducted in each area. The researcher used judgmental sampling to select respondents for the FGDs. In judgmental sampling, a cross-section of the sample selected by the researcher conforms to representative sample [28]. This type of sampling is more common in qualitative research [29]. Open-ended questions were used to collect the data. An interview guide for the focus group was designed after a thorough literature review. The interview guide includes questions like what the changes they have observed before and after the cyclones Sidr and Aila are, how the children, women, and older adults cope with such climate change related disasters, how Sidr/Aila affected their livelihood patterns, and so forth [1].

The qualitative data were collected by using a focus group discussion (FGD) from the following two groups of respondents.

*Group 1*. It consists of knowledgeable (educated) people from upazila and union level (a union on average has 20,000 people). The target participants were NGO staffs, schoolteachers, school headmasters, social workers, community leaders, and local government officers. 

**Figure 2:**
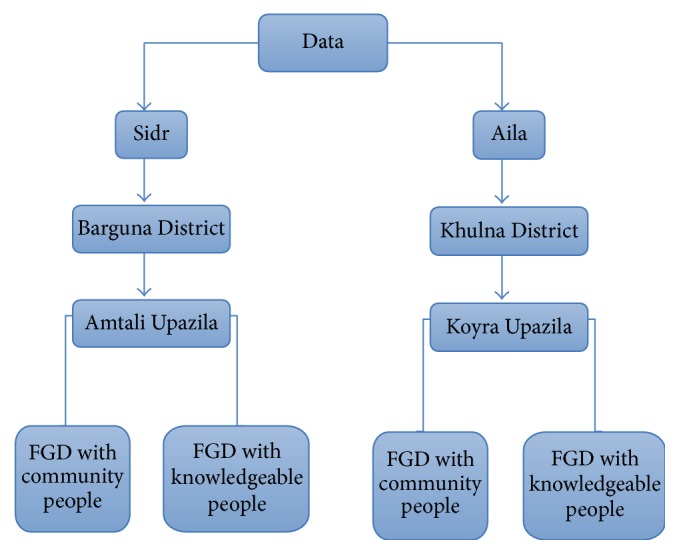
Data collection process.

*Group 2*. It consists of members of the local community, which included former UP (Union Porishod) member, farmers, businessmen, fishermen, community members, housewives, and a current UP member (Figure 2).

In order to make the best use of the data, this research employed a thematic analysis of the focus group discussion; texts were extracted with the aim of evaluating the affected coastal peoples' experiences of climate change. To analyse the data, the researcher followed the six phases of analysis as suggested by Braun and Clarke [30]. In the first phase, the transcripts of the focus groups were read several times to obtain a sense of the whole. The researcher carefully went through the descriptive responses given by the respondents to each question in order to understand the meaning they had communicated in the focus group discussions. During this phase, notes were taken to generate an initial list of ideas (Table 1).

**Figure 3:**
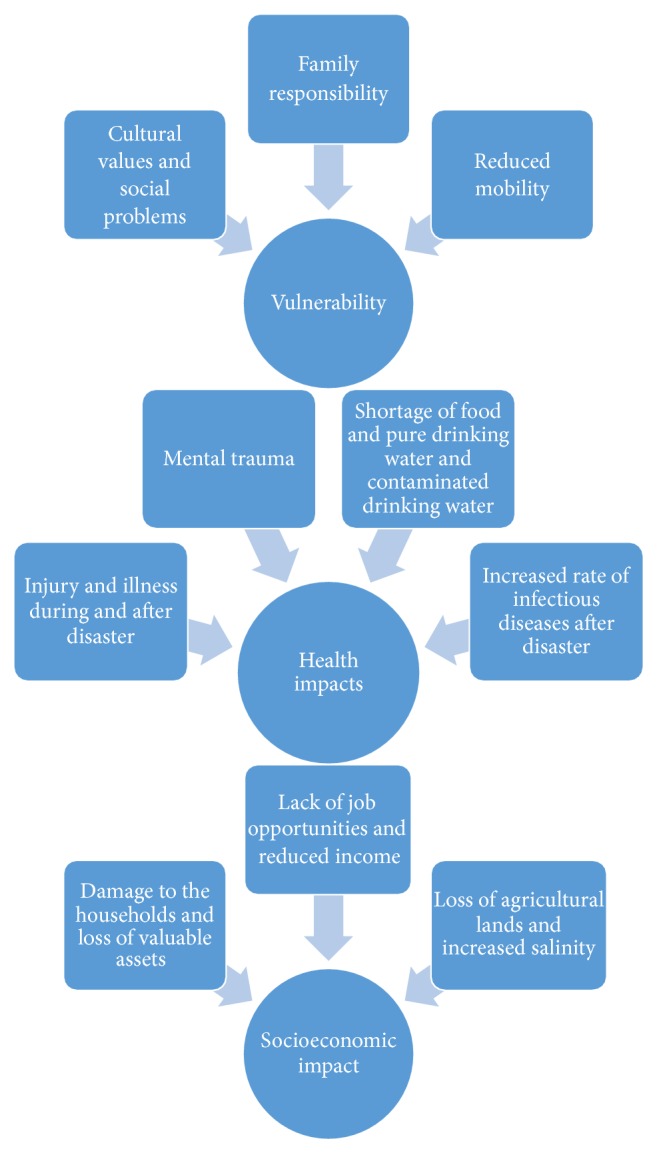
Thematic map, showing three main themes.

In the second phase, codes were developed from the data to identify key themes. After all the focus group discussions had been coded once, codes were then refined to capture the concealed meaning. In phase four, the researcher reviewed and refined the emerged themes. The researcher went through all the extracted data for each theme and made sure that the extracted data was appropriate to the theme. Information that did not fit into any theme was excluded. A thematic map was developed and all the themes were given a name to identify the essence of what actually had been captured from the data in phase five and the last stage is analysis (Figure 3).

## 3. Findings

The following three themes emerged from the data analysis. The first theme explores the impact on the socioeconomic status of the people in the affected areas. The second theme presents the impact on health. Lastly, the third theme focuses on the vulnerability of people.

### 3.1. Impact on Socioeconomic Status

According to the respondents, for the past ten years or more significant changes have been observed in the surrounding environmental conditions including the weather and climate of the coastal areas. Natural disasters like cyclones, storms, and flooding frequency have increased in the area a lot more than previous times. The respondents of the study pointed out that two consecutive cyclones, “Sidr” and “Aila,” brought noteworthy changes in their living pattern. People are struggling to go back to their previous quality of life prior to Sidr and Aila. After the cyclones in both the villages, dams were damaged and sea saline water came to the agricultural lands easily. Salinity in the water has increased which has affected the production of crops and agricultural lands are not suitable for growing crops anymore. Most of the residents (in both villages) earn their living by fishing and farming agricultural lands. Production of agricultural products in these lands reduced remarkably after the disaster hit the area and now these farmers are suffering financially. One of the respondents from Borobari Village stated that

*before Aila hit the area, they used to produce a lot of different agricultural goods including fresh vegetables and fruits. By selling them in the mainstream market they could have earned the money that was sufficient for them to live a year on without any further production, but today this is not possible and also nature is not supporting them.*

The respondents experienced that salinity in the coastal area has become severe and saline water mixed with cultivated lands. As a result, cultivated lands are becoming abandoned. Due to high salinity, cattle were not getting enough food. This led to farmers finding it extremely hard to use them for ploughing the farming land(s). Poor people who used to cultivate crops and graze their cattle on the open space of shoals said frequent disasters are taking their toll on the agriculture sector forcing them to change their cultivation pattern. Many sweet water fish died due to the influx of saline water while water hyacinth and crops were rotten.

Further to these challenges in agriculture, climate change has also had harmful effects on other community sources of livelihoods such as casual labouring, fishing, hunting, and crafting. It is worthy to note that many of these livelihoods depend on the performance of agriculture; for example, casual labouring is usually done on weeding and harvesting.

Fishing boats were wrecked after the cyclone. Though fishermen along with other affected population in the villages received some sort of financial support from the government organizations (GOs) and nongovernment organizations (NGOs), the financial assistance was not adequate enough for them to buy fishing boats. Some of the families, who lost everything due to cyclones, used the money for reestablishing their living place and meeting their daily needs. One of the Union Porishod (UP) members of the Borobari Village said:

* “We live our life like a wanderer amid insecurity and thwarting as sometimes our crops lands are destroyed by cyclonic storm.”  One of the respondents from Balitali Village of Amtali Upazila said in a depressed tone, “Me and my family are living a wretched life and have been struggling with poverty since becoming landless.”*

Just after the disaster, there was a severe shortage of food and scarcity of pure drinking water in the affected areas. Though some families managed to get some food due to a lack of combustible substances, cooking was not easy and they had to live day after day eating raw food. Most of the local food shops were damaged or closed due to a lack of supplies (the lack of supplies being a result of the breakdown in the transport system). Nongovernment organizations again provided some relief but government help was not adequate.

Most of the households have poor housing conditions that are highly vulnerable to natural disasters. Prior to Sidr and Aila, the financial situation of the community in the affected villages was not great; however, after Sidr and Aila, it deteriorated even further. Their living conditions are now poor; however, many GOs and NGOs are working to bring back normalcy. There are not enough job opportunities in the local areas and those who were farmers and fishermen are also trying to switch their jobs in order to increase their finances. One of the respondents from the Aila affected region reported,

*Post Aila, every household is going through mental pressure and anxiety. They are devastated and discarded by losing their agricultural land, households, livestock's, fishing ponds and so on. People of these areas are still working hard to get back to their normal lives. Some of the families started a new life by taking loans from local non-government organizations, but some failed to pay the repayments because frequent disasters made it difficult for them to grow crops. This put them under immense mental pressure.*

The headmaster of Bedkashi Borobari Government Primary School expressed that

*it is very unfortunate that the level of education in the area is not satisfactory as the education rate is too low here. Most of the parents do not send their children to the school due to poverty. Their only choice is to send children to work in order to make living. *

Another teacher from the same school said the student drop-out rate is alarming and more girls are dropping out than boys. He also pointed out that distance is a factor for children not continuing education in this area. Families that suffered hugely after disaster are engaging their children in household activities and forcing them to work and earn money for the families so these children were dropping out from schools.

One of the respondents who was severely affected by cyclone Aila stated that, due to a lack of job opportunities and to maintain the household expenses, some of the families and in some cases the heads of the households are leaving the village and migrating to different cities like Dhaka, Khulna, and Chittagong. Some people had migrated to the neighboring country India. Another respondent shared that

*many people like me go to the deep forest in the Sundarban to collect honey, crab and fishing to meet our family expenses. It is indeed a risk of life to do this kind of job, as there is a fear of being attacked by the Royal Bengal Tigers and pirates. Hence our life is uncertain, as we have to risk of our life to do this. Once we go out for our job, our family members also worry for us and keep praying until we come back. This really is a huge mental pressure which makes our life miserable….*

People are leading a very miserable life as their income from agriculture and fishing, their only means of livelihoods, is just insufficient. This resembles a larger picture of Bangladesh where climate change has threatened the lives and livelihoods of millions of people.

### 3.2. Impact on Health

The FGD explored information about recent environmental changes due to climate change and the disaster impact on the health of the coastal population. Specifically, the main aim was to get information from the participants about the impact on health (such as general health situation, mental health, and wounds) during Sidr and Aila and after Sidr and Aila. In order to understand the magnitude of the problem, information on the health condition of people in the community before the natural disaster was also gathered. Changes observed and experienced by people in relation to climate change were also expressed by respondents.

When respondents were asked “what does climate change mean to you?” they explained the change in the weather condition is climate change to them. Some replied that the increased frequency of natural disasters, temperature rise, and sea level rises are all signs of the changing climate in their area.

Chairman of Amtali Upazila stated,

* Seasons are changing, its more hotter during summer, winter comes very late and stay for a short period, lands are not suitable for farming anymore, crops growing has reduced, natural disasters hit repeatedly and erratic rainfall are happening due to climate change.*

Participants believed that the climate is changing and these changes are not bringing anything beneficial for them. They have been living in these areas for many years and in the last 10 years or more they have witnessed a significant difference in current climatic conditions. Since they are living close to the coast, the changes they have observed are a sea level rise, frequent occurrence of natural disasters like storms, cyclones Sidr and Aila, and floods, influx of saline water in the agricultural land, excessive temperature rises during summer time, and irregular patterns of rainfall. All these changes are making a substantial impact on their living pattern and health.

Participants were asked about the impact of climate change on health. A number of participants gave their opinions on the issue and they included the following: during and after the cyclone, people in the affected villages suffered from various diseases including diarrhoea, dysentery, viral fever with cough, cold, skin diseases, eye infections, pain, paralysis, jaundice, lack of nutrition, waterborne diseases, typhoid, anemia, high blood pressure, and severe headaches (most common).

One respondent explained,

*Climate change is responsible for Sidr in our area. The saline contaminated and polluted water is responsible for spreading the communicable diseases in the area. *

A lack of pure drinking water also aggravated the spread of waterborne diseases in the affected areas. According to the participants, the main sources of the infectious diseases are polluted and highly saline water, unhealthy sanitation, unclean environment, unhygienic food, excessive hot weather, and vector borne infections. Due to climate change, natural calamity is now common in these areas. This changes the natural environment and more infectious diseases are emerging (many of which were not common 20 years ago).

The prevalence of diarrhoea, skin diseases, typhoid fever, and jaundice rapidly increased just after Sidr. Local health services lack the manpower and resources to deal with the variety of health problems. During emergency situations, this is even more evident. Many of the poorer people have travelled to the nearest town to get better treatment.

The Chairman of Amtali Upazila said,

*Children and other age group people mainly suffered from diarrhoea and jaundice, but he doesn't know if anyone died of diarrhoea or jaundice.*

Diarrhoea, typhoid, and skin diseases spread rapidly due to the infected water that resulted from cyclone Aila. Diarrhoea and skin diseases were the most common problems identified by the respondents. Participants could not provide any mortality data from the occurrence of diarrhoea in the area but in each household in the village there were cases of diarrhoeal disease. There were also some cases of amoebic dysentery and bloody dysentery reported in the discussion.

It has been found that “*diarrhoea, typhoid, and skin diseases were present like other normal diseases before Aila. After Aila, excessive polluted water and a shortage of a drinking water supply meant that the majority of the families suffered from these diseases.*”

When they took shelter in the shelter centre, these diseases started spreading very quickly. There were some cases of respiratory tract infection. A lack of sufficient food and absence of a nutritious diet in the shelter centre forced people to starve. Eventually, they became weaker and more prone to infectious diseases. As a result, they could not work properly on their land. Some of them were farmers who became so seriously ill after the cyclone that they sold their agricultural land(s) to maintain the cost of their treatment. It emerged from the discussion that there are some people in the village who lost everything during the cyclone. Some of them became mentally unstable and many families lost their children (bodies recovered after the cyclone). Some of them could not bear the loss of their near ones and were traumatized psychologically.

One of the participants shared that

*some of the village women got fainted and lost their mental stability by just seeing the devastation caused by the cyclone Aila in the village and some of them are still recovering from the shock and can't forget the incident from their mind.*

Efforts were made to assess the impact of disasters on injuries. Many mentioned that injuries were common during the disaster time. Many people got injured during the cyclone. In Amtali, many people were struck by tin (used to build houses in rural areas), fell under trees, broke their legs, and had cuts and bruises on different parts of the body. Injuries to the head and spine were also common. A high number of people were wounded. During Sidr, the high volume of water washed people away. Boats and other hard objects wounded many. Currently, there are many wounded persons who are still suffering from different kinds of side effects. Many are now disabled due to the injury sustained during the cyclone.

To tackle the health problems, both GO and NGOs are working in the affected areas. They introduced a new deep tube well for safe drinking water, constructed hygienic toilets, and distributed water-purifying tablets; bleaching powder and awareness building programmes were undertaken by different agencies. They also distributed general medicines to the people in the community.

When questioning the current general health situation of the community, all the participants agreed that the current health situation is reasonably better now than during and immediately after the disaster. The health situation has improved because of different programme interventions by different local and international agencies. According to the participants, the health situation is improving.

### 3.3. Impact on the Vulnerable Population

When the discussion moved on to the vulnerable population in relation to the cyclones Aila and Sidr, the respondents described the elderly, infants, and women as the most affected by the disasters. They endured a difficult time at the shelter centre after the cyclones. Children experienced malnutrition due to a lack of food, which subsequently affected their physical and mental growth. School children could not complete their education on time. Many children also suffered from diarrhoea, dysentery, and skin diseases. Elderly people were found with cuts and bruises on hands and legs, broken hands and legs, bruises on their head, and injuries to different parts of the body.

Many elderly people also died due to falling trees and houses (because of their reduced mobility). In some cases, elderly people were found under traps while trying to save the lives of others. Both usually were found dead at the same place. Those that survived the adverse conditions now find that their health is deteriorating each day; however, no exact figure of the number of children and elderly people affected was given. Representatives of the GO and NGO also could not recall the precise statistics. Some households did not take any shelter because they were physically unable to reach the shelter because of the adverse condition or because it was too far. Some of them did not understand the significance of the warning or could not hear the warning. They decided to stay at home because they were fearful that the house would be looted. Many people thought that the shelter centres would be unsafe and unpleasant.

One of the Union Porishod members of Sidr affected village reported,

*…it's the women who suffered a lot during the disaster time due to a lack of preparation. Many women couldn't ran away because their clothes got stuck on trees. Many of them died after trees fell on them.*

The majority of the women of the coastal areas are homebound and when the cyclone struck they were busy with family livestock and had to make suitable provision prior to going to the shelter. In many cases, it was found that the traditional clothing “Sari” (that women wear in the villages) was responsible for hindering their escape from the cyclone when entrapped with some objects while running. Women were also responsible for the children of their families. In many cases, it was noted that children could not evacuate quickly enough and mothers, while attempting to save their children, died during the cyclones. According to the respondents, most of the women of the households are dependent on their husbands for taking any decision. When the cyclone information came out, it was not available to them and in some cases they had little information. All of these factors contributed to them not evacuating the house and put them in more danger during the cyclone.

Cochairman of Amtali Upazila shared an incident that he had witnessed:

*When the cyclone hit the area, a pregnant woman died because the transportation system had broken down in the next town.*

Pregnant women especially went through immense mental and physical trauma and it was later found that many of them gave birth to disabled children. The interviewees described how women were also harassed and abused while they tried to collect food and relief materials for their families. Many of them fainted upon witnessing the severity of the cyclone. Women and teenage girls who stayed temporarily in the shelter centre could not use the toilet facility because of the lack of privacy and lack of sanitation products. This caused them to suffer physically and mentally. Mothers could not find a private place to breast-feed their children and they could not take care of children properly, thus creating a social problem. Many families, specifically those who are headed by females, had to fight even after the cyclones to collect the aid as it involved dealing with dictatorial males and they had little experience of this.

Generally, the women from each household walk a fair distance to collect pure drinking water for their families. When the dams broke during the cyclone and salinity increased, they had to struggle more to get water (which affected them physically). Often mothers starved both days and nights. Any food they managed to get they gave to their children and other family members causing their own physical health to suffer. Most of the women's families broke down when the impact of the cyclone worsened their financial conditions.

The UP member of Koyra Upazila said that

*many of the women divorced when they lost their home or assets to the impact of cyclones. *

Some of the middle aged women work as a daily domestic help in the houses of affluent people (there are only a handful in the entire village). By doing this, they can get some food and money but when there is no work, they have to remain half-fed or unfed. Women of these affected villages are suffering severely from anemia, leucorrhoea, infertility, and irregular menstruation (as reported by the NGO people who were present in the discussion).

## 4. Discussion and Conclusion

It is quite evident from the literature that human actions are responsible for changing the global climate and it is increasing at an alarming rate. The impacts of climate change have been a major topic of discussion for researchers and scientists. As climate change proceeds, the frequency and intensity of extreme weather events such as cyclones, heat waves, flooding, droughts, and heavy precipitation are going to increase noticeably. Although the global frequency of tropical cyclones is expected to decrease or remain essentially unchanged, they may become more intense.

From the focus group discussion with the local community people and knowledgeable people in the community, three themes were generated in this research about the effect of the cyclones. The themes are the impact on socioeconomic status, the impact on the health of the people, and lastly the impact on the vulnerable population. The first theme on socioeconomic status reveals that recent cyclones Sidr and Aila brought noteworthy changes in their living pattern. People are struggling to go back to their previous quality of life. Most of the residents (in both villages) earn their living by fishing and farming agricultural lands. Production of agricultural products in these lands reduced remarkably after the disaster hit the area and now these farmers are suffering financially. These findings are in keeping with other studies such as Furberg et al. [5], Wei et al. [2], Guha-Sapir et al. [3], and Srikanthan [4].

The second theme discussed the impact on health of the coastal people. Respondents believed that the climate is changing and these changes are not bringing anything beneficial for them. A lack of pure drinking water aggravated the spread of waterborne diseases in the affected areas. According to the participants of this research, the main 249 sources of the infectious diseases are polluted and highly saline water. An extreme weather event reduces access to safe drinking water and food and increases the chances of having infectious diseases among the vulnerable pregnant women. A lack of pregnancy care further complicates their health conditions [31].

The analysis shows that mental health problems are also a major growing concern for the climate-affected population. The post-*Sidr *and post-*Aila *results show that the number of people with mental health problems has increased dramatically.

The third theme explored the most vulnerable population from the climate induced natural disasters. The analysis concludes that women, elderly people, and children are the most vulnerable groups of people in the community. The kind of clothing women wear causes a major obstruction in their mobility and quick recovery during an emergency situation becomes hard for the vulnerable women. These findings are similar to those in research carried out by Mehta [32] and Dasgupta et al. [33]. Climate change affects both men and women; however, the impact is felt more by women. Climate change increases the females' burden of meeting their household responsibilities. During cyclones, children and women are more vulnerable because of their physical formation. Most of the females have a low level of education. Women may have been more at risk because they stayed behind to save or search for their children, while others escaped. Results from this research resonate with other research studies by Adebo and Sekumade [6], Adeniyi et al. [7], and Guha-Sapir et al. [3]. Children and the elderly were found most vulnerable during the cyclone. They are vulnerable due to health and physical vulnerabilities and because of their inability to run fast. Children are also suffering from health problems such as malnutrition. These results are similar to other studies by Devkota et al. [8], Kendrovski and Spasenovska [9], Davies et al. [10], and Bhuiyan and Khan [11].

The affected population of cyclones Sidr and Aila are still facing huge problems due to adverse impacts of climate change and cyclones also have increased the vulnerabilities of these people. Climate change related natural disasters damage livelihood of poor people and make them more vulnerable.

This study has exposed three major impacts of climate change through natural disasters on the coastal people. Climate change makes people's life a difficult one and it increases vulnerability in the society such as poor people, children, the elderly population, and women. Geographically, Bangladesh is located in the danger zone and the country is more vulnerable to natural disasters like cyclone and flooding every year and this study will address the coping strategy and new policies of adaptation for the vulnerable population in the coastal areas.

Proper physical measures can be taken by Bangladeshi government to prevent these vulnerable people from future catastrophic disasters. Stakeholders and policymakers should take the initiative to develop skills and experience to analyse the changing climate and to identify the causes for change and assess the health effects. To reduce the vulnerability of costal communities, capacity building programmes should be run at household levels to adapt to climate change impacts and different livelihood strategies. Adaptation measures at community level will help local people to strengthen their barriers against climate-related disasters. There is limited research on understanding the likely health impacts of climate change through natural disasters and also understanding the extent to which climate change will affect the health of the general population is underresearched. The research gaps and other information provided in this research lead to a great number of potential research ideas.

## Figures and Tables

**Table 1:** Data extracted with codes and themes applied.

	Data extracted	Coded for	Themes
FGD with community people at Sidr affected areas	The incidence of diarrhoea and skin diseases increased after cyclone Sidr. Though diarrhoea was in control, the cases of skin diseases were very high (Respondent, Baliatali Village).	(1) Diarrhoea and skin diseases	Impact on health
FGD with health and service providers and other knowledgeable people	There were many cases of diarrhoea and typhoid just after the cyclone hit the area; however, people in this village mainly suffered from skin diseases (Union Porishod Chairman, Baliatali Village).	(1) Diarrhoea and typhoid (2) Skin diseases	
